# A mixed-methods study of body esteem, disordered eating behaviors, acculturative stress, and sociocultural correlates among female Chinese international students

**DOI:** 10.1186/s40337-026-01599-6

**Published:** 2026-04-04

**Authors:** Peiyi Wang, Wenqi Zhang, Chuansheng Chen, Ilona S. Yim

**Affiliations:** 1 Department of Psychological Science, University of Texas Rio Grande Valley, ELABN 356, 1407 E Freddy Gonzalez Dr, Edinburg, Edinburg, TX 78542 USA; 2 Department of Psychology, University of California, Irvine, USA

**Keywords:** Cultural transition, Acculturative stress, International students, Body esteem, Eating behaviors, Tripartite influence model

## Abstract

**Background:**

Global migration has heightened the need to understand how cultural transitions influence body image and eating behaviors. In the U.S., Chinese international students represent one of the largest migrant student groups, with women showing particular vulnerability to body image concerns and disordered eating. Yet, the ways in which cultural transition and acculturative stress shape these outcomes remain insufficiently studied.

**Methods:**

This study investigated female Chinese international students using online surveys with free-response questions administered during researcher-supervised video sessions prior to their migration to the U.S. for college (*n* = 127) and again six months after arrival (*n* = 113; 89% retention).

**Results:**

Across time, participants reported lower body esteem, higher emotional eating, and higher BMI, while perceived sociocultural pressures from different sources and internalization of different body ideals remained stable. Acculturative stress was associated with greater muscular-ideal internalization, lower body esteem, and higher uncontrolled eating at follow-up, even after accounting for pre-migration levels. Qualitative analyses provided additional context, with participants describing shifts toward muscular/fit ideals in the U.S. that shaped body image concerns, dissatisfaction with food environments that fueled eating concerns, and other experiences influencing body image and eating behaviors.

**Conclusions:**

By integrating quantitative and qualitative evidence, this study contributes to the limited research on international migrants and underscores the associations between acculturation experiences, acculturative stress, body image, and eating. Findings highlight food environments and cultural ideals as complex factors within acculturation and tripartite influence models and point to actionable strategies for policies and interventions supporting international students during cultural transition.

## Abstract (plain language summary)

Migration can heighten risks for body image disturbances and disordered eating, partly due to new sociocultural appearance pressures and the stress of cultural transition. However, little is known about how cultural transition and acculturative stress shape these changes. This mixed-methods study examined body image and eating behaviors from pre-migration to six months after female Chinese international students arrived in the United States for college. Quantitative results indicated significant decrease in body esteem and increases in emotional eating and BMI across time. Acculturative stress was uniquely related to greater muscular-ideal internalization, lower body esteem, and higher uncontrolled eating at follow-up. Qualitative findings corroborated these patterns and offered deeper context. Students described shifting toward U.S. muscular/fit ideals, heightened self-comparison, and dissatisfaction with food environments that disrupted eating patterns. Additional experiences such as navigating cultural norms around appearance further shaped body-related well-being. These findings underscore acculturative stress as an important mechanism linking migration to body image and eating vulnerabilities. Prevention and campus-based support efforts for international students may benefit from addressing cultural adjustment challenges, providing more varied food options in dining halls, and fostering healthier body-related cognitions during early migration.

Migration can negatively impact body image and eating behaviors partly due to exposure to differing sociocultural pressures regarding physical aesthetics and the stressful experience of change [1–5]. In parallel, prevalence rates of body dysmorphic disorder and eating disorders have risen among college-aged individuals worldwide in recent years, including in China [6, 7] and the United States [8, 9], and are associated with substantial adverse consequences for psychological adjustment and physical health, including suicidal ideation and attempts [10]. Despite the multidimensional etiologies of body image and eating-related concerns [11, 12], the body image and eating behaviors of international migrants (i.e., individuals born outside their country of residence), particularly in relation to cultural transition experiences, remain understudied [13]. Chinese international students, who represent one of the largest international student groups in the U.S [14]., go through the challenge of navigating different aesthetic standards between Chinese and U.S. cultures [15–17] while experiencing acculturation-related stress [i.e., acculturative stress; 18]. Female young adults, in particular, face disproportionately higher and unique vulnerabilities to body image and eating concerns compared to other gender identities, although all genders are susceptible to these issues [19]. To address current research gaps and enhance the diversity of body image research, this study aimed to quantitatively examine changes in perceived sociocultural pressures toward body ideals (from family, peers, significant other, and media), body ideal internalization, body esteem, and eating behaviors among female Chinese international students. Furthermore, it was aimed to qualitatively understand how cultural transition and acculturative stress influence their body image and eating behaviors.

One of the most widely applied sociocultural frameworks of body image and eating disturbances is the Tripartite Influence Model [20]. This model proposes that three sources of sociocultural pressures (parents, peers, and media) toward specific body ideals contribute to body image concerns through body ideal internalization and appearance comparisons, which in turn increase vulnerability to eating disturbances [20]. Subsequent extensions have broadened the sources of sociocultural pressures by adding significant others [21–23], and expanded body ideal internalization to encompass not only specific body shape ideals such as thinness and muscularity, but also general attractiveness (i.e., the concern with general appearance and the desire to be physically attractive in general) [24, 25]. Although these predictors were initially identified in research with White female samples in Western contexts, emerging evidence supports their applicability in diverse populations. For example, one study among Chinese adolescent girls confirmed the role of sociocultural pressures, thin-ideal internalization, and appearance comparison in predicting body image and eating behaviors [26]. Nevertheless, most existing research has focused primarily on thinness-related pressures and internalization. Importantly, no studies have investigated how these predictors, across multiple sources of sociocultural pressure and various body ideals, change over time among international migrants, despite the critical implications for understanding their dynamic role in body image and eating behaviors.

During cultural transition, perceived sociocultural pressures may remain stable or shift depending on their source, and different body ideals may be internalized to varying degrees. Studies of female Chinese international students suggest that family influence often diminishes as students gain independence and move away from home, while exposure to new societal expectations introduces additional appearance-related pressures [27, 28]. At the same time, reliance on the internet and communication with family during the adjustment period may sustain or even reinforce family pressures related to body image [29], making the direction of change less clear. Peer influence may intensify in the U.S. college environment, where students spend substantial time with same-aged peers, and where peer norms play a central role in international students’ social adjustment [30]. For Chinese international students, however, peer influence is likely to be multifaceted, as they interact with diverse groups of peers who may differ in their body image preferences and in the openness with which body-related topics are discussed [31]. Similarly, the role of pressures from significant others remains largely unexplored in this population, making it essential to examine these influences. Media influence, however, is likely to remain relatively stable, as international students typically maintain access to both Chinese and Western social media platforms, which continue to disseminate appearance-related ideals [32].

With respect to body ideals, research indicates that muscular-ideal internalization tends to increase during cultural transition, driven by consistent evidence of greater exposure to athletic and fit ideals in Western media and peer context [33–35]. In China, a “pale, young, and slim” ideal, reflecting preferences for lighter skin tone, youthfulness, and thinness, has been documented as a prevalent body ideal [36]. In contrast, among U.S. undergraduates, the perceived ideal female body increasingly combines thinness with muscularity, with women expressing greater preference for a “muscular thin” body type compared with a solely thin figure [35, 37]. However, the trajectories of thin-ideal and general attractiveness-ideal internalization appear more complex. Thin-ideal internalization may attenuate in the U.S. context, where body positivity movements are increasingly visible and challenge dominant thinness norms [38–40]. Yet studies of Asian Americans including diverse descents such as Chinese and Korean suggest that thinness continues to be highly valued among this group [41, 42]. Also, the internalization of a general attractiveness ideal remains insufficiently understood and warrants further exploration. Given the cultural and contextual complexities of these processes, a mixed-methods approach is necessary to capture both quantitative patterns of change and qualitative insights into students’ lived experiences.

Acculturative stress is a critical construct in understanding migrants’ health. It arises when the challenges of cultural adaptation, such as navigating mainstream American culture while maintaining one’s heritage culture, exceed an individual’s coping resources [18, 43]. Chinese international students commonly experience acculturative stress in the form of language barriers, intercultural conflicts, feelings of inferiority, cultural isolation, perceived discrimination, and limited work opportunities [44–48]. Acculturative stress has been identified as a key factor associated with poorer psychological well-being and adverse health outcomes among racial and ethnic minorities in the U.S [49]. Given the greater cultural distance between Chinese and American cultures, Chinese international students report higher levels of acculturative stress than their Hispanic and European counterparts [50, 51]. Within this group, acculturative stress has been linked to elevated depression [48] and lower quality of life [52].

Poor body esteem and problematic eating behaviors may be closely related to the experience of acculturative stress. To date, however, no studies have specifically examined these associations among Chinese international students. In ethnic minority families, slender bodies and Westernized features are sometimes believed as a means of overcoming cultural adaptation challenges and achieving social mobility in U.S. society [53]. As racial and ethnic minorities navigate mainstream culture, they may also struggle with the devaluation of ethnic physical traits (such as eye shape among Asians in general), which can further undermine body esteem [54]. Empirical work among Hispanic and Asian young adults of diverse subgroups (e.g., Chinese, Vietnamese) shows that acculturative stress is associated with body image disturbances [55, 56].

With respect to eating behavior, theoretical frameworks also suggest important links with acculturative stress. Escape Theory posits that individuals may engage in uncontrolled eating to distract themselves from aversive self-awareness and distressing thoughts [57]. Consistent with this perspective, problematic eating among ethnic minorities has been understood as a coping mechanism in response to both racial oppression and acculturative stress [58]. For Asian populations (e.g., Chinese Americans or Chinese nationals), such behaviors may be reinforced by cultural norms emphasizing emotional restraint, which can promote maladaptive strategies for managing stress [59, 60]. Supporting this view, empirical studies have found that acculturative stress predicts higher uncontrolled and emotional eating among Asian young adults, predominantly of Chinese descent [61], while qualitative interviews with racial and ethnic minority women identify acculturative stress as a key contributor to binge eating problems [62]. Furthermore, Chinese immigrant individuals reported that there are drastic differences in food choices, and they struggle with diet acculturation in the U.S [63, 64]. This might lead to problematic eating behaviors among this population. Despite these converging lines of evidence, no studies have examined the relationship between acculturative stress, body esteem, and eating behaviors among female Chinese international students undergoing cultural transition.

## The current study

This mixed-methods study examined how key factors identified in the Tripartite Influence Model change with cultural transition and whether acculturative stress is associated with these factors among female Chinese international college students. Data were collected at two time points: before students moved to the U.S. for college (T1) and six months after arrival (T2), allowing examination of changes associated with cultural transition.

The first aim was to quantitatively assess changes in perceived sociocultural pressures (family, peers, significant others, media), internalization of body ideals (thinness, muscularity, general attractiveness), body esteem, and eating behaviors (uncontrolled eating, emotional eating, and cognitive restraint) from T1 to T2. It was hypothesized that perceived pressures from media would remain unchanged, and the internalization of muscular ideal would increase. Body esteem was expected to decrease while problematic eating behaviors were expected to increase. For other variables, given limited and mixed prior findings, no specific hypotheses were advanced. The second aim was to examine whether acculturative stress at T2 was uniquely related to body esteem and eating behaviors at T2 after accounting for baseline levels. It was hypothesized that higher acculturative stress would be associated with lower body esteem and higher problematic eating behaviors six months post-move. The third aim was to qualitatively explore participants’ perspectives on how cultural change influences body image and eating behaviors, providing contextual insights that complement the quantitative findings.

## Method

### Recruitment and procedure

A prospective longitudinal cohort survey design incorporating both quantitative and qualitative components was used to address the research questions. Recruitment took place across online Chinese social media platforms (the RED app and WeChat public accounts) and in-person events from June to September 2023. Data collection occurred at two time points: Time 1 (T1), between July and September 2023, before the start of all students’ first semester/quarter, and Time 2 (T2), between February and April 2024, approximately six months after they began their studies in the U.S. The six-month interval was selected based on general patterns of cultural adjustment and learning among international migrants [Chapter 7; 65] and aligns with prior studies on international students demonstrating changes in psychosocial variables [e.g., 66, 67].

For recruitment, once a potential participant contacted the research team after seeing the advertisement, eligibility (see the Participants section) was confirmed, an explanation of the study procedure was provided, and an online session for consent and T1 data collection was scheduled. All data collection was conducted through real-time online video conference sessions. During the T1 session, the researcher reviewed the study information sheet, obtained online written informed consent, and assigned a participant ID. The T2 session was also scheduled at this time. Participants were sent a link to the online questionnaire, along with their participant ID, and a researcher remained available online to answer any questions, ensuring data quality. The T2 data collection process was similar to T1, except participants did not need to sign informed consent again.

Each anonymous online questionnaire took approximately 30 min to complete. The entire study was conducted in Mandarin Chinese to control for the potential impact of language on culture-related experiences [68–70]. Participants received a $5 gift card upon completing T1 participation, and those who completed the T2 questionnaire received a $10 gift card. The study received IRB approval from the University of Californian, Irvine (IRB: #2516). The deidentified quantitative data is available online [71].

### Participants

Eligible Chinese female international students met the following criteria: (1) 18 years or older, (2) self-identified as female, (3) accepted an admission offer from a university in the U.S., (4) considered themselves international students, (5) held Chinese citizenship, (6) were freshmen in the Fall 2023 quarter/semester, and (7) considered Mandarin Chinese as the native language. Those with a history of, or currently experiencing, or treating, eating disorders were not eligible to participate.

A total of 127 participants were recruited for T1 data collection, with an 89% retention rate (*n* = 113) for T2 data collection. One participant reported current experience and treatment for an eating disorder in their survey response, leading to the post hoc removal of the corresponding data. As a result, 112 participants were included in the final analyses. This sample size was sufficient to detect a small-to-medium effect size (*d* = 0.30) with 88% power for paired-sample t-tests and 86% power for hierarchical regression analysis at an alpha level of 0.05, as calculated using G*Power [72].

### Measures

#### Perceived pressures on body image from family, peers, significant others, and media

The validated Chinese version of the Sociocultural Attitudes Towards Appearance Questionnaire-4-Revised (SATAQ-4R) scale was used to measure perceived pressures on body image from sociocultural sources, including family, peers, significant others, and media [25, 73]. An example question assessing perceived pressures from these sources is, “I feel pressure from [the source] to improve my appearance.” Each source of pressure was assessed using four items, rated on a five-point Likert scale ranging from 1 (*definitely disagree*) to 5 (*definitely agree*). Higher scores indicate greater perceived pressures.

In the validated Chinese version, the Pressure from Family, Peers, & Significant Others (Cronbach’s alpha = 0.93; 12 items) emerged to be one factor and Pressure from Media (Cronbach’s alpha = 0.95; 4 items) as the other factor for Chinese female college students [73]. In the current sample, Cronbach’s alpha levels for the two subscales were 0.88 and 0.89 at T1 and 0.91 and 0.92 at T2.

#### Internalizations of thinness, muscular, and general attractiveness ideals

The same scale (SATAQ-4R; Schaefer et al., 2017; Wu et al., 2020) was used to measure internalizations of thinness, muscular, and general attractiveness ideals. Each subscale was measured with multiple items rated on a five-point Likert scale ranging from 1 (*definitely disagree*) to 5 (*definitely agree*), with higher scores reflecting greater internalization. Example items include: “I want my body to look very thin” (thinness ideal), “It is important for me to look muscular” (muscular ideal), and “It is important for me to look good in the clothes I wear” (general attractiveness ideal).

In the validated Chinese version, the Internalization of Muscular Ideal was one factor (Cronbach’s alpha = 0.79; 5 items) and the Internalization of Thinness & General Attractiveness Ideal was the other factor (Cronbach’s alpha = 0.85; 10 items) for Chinese female college students (Wu et al., 2020). In the current sample, Cronbach’s alpha levels for the two subscales were 0.89 and 0.84 at T1 and 0.91 and 0.84 at T2.

#### Acculturative stress

The Riverside Acculturative Stress Inventory [15 items; 74] was used to measure acculturative stress post-migration at T2. This scale was chosen for its non-ethnic-group-specific nature [75], making it applicable to the Chinese international student population, and consistent with the acculturation theory [18]. The acculturative stress domains and example questions are as follows: Intercultural Relations (e.g., “I feel that my particular Chinese practices have caused conflict in my relationships.”), Cultural Isolation (e.g., “When I am in a place or room where I am the only Chinese, I often feel different or isolated.“), Work Challenge (e.g., “In looking for a job, I sometimes feel that my Chinese background is a limitation.“), Language Skills (e.g., “It’s hard for me to perform well at work because of my English skills.“), and Discrimination (e.g., “I have been treated rudely or unfairly because of my Chinese background.“). Each item was rated on a 5-point Likert scale (1 = *strongly disagree* to 5 = *strongly agree*), with a higher score representing a greater level of acculturative stress. The use of the overall score is recommended [76]. The reliability for the overall scale in the original scale development study was .83 among college students [75], and .76 in this study measured at T2.

#### Body esteem

The Appearance and Weight subscales of the Chinese version of the Body Esteem Scale [77] were used to measure body esteem, following previous studies [78–80]. The Appearance domain measures satisfaction with physical appearance, including facial features and body shape, using 10 items. A sample question for the Appearance domain is, “I’m pretty happy about the way I look.” The Weight domain measures satisfaction with body weight using 8 items (e.g., “I really like what I weigh.”). Each item was rated on a 5-point scale ranging from 0 (*Never*) to 4 (*Always*). Higher scores indicate greater body satisfaction. This scale has demonstrated high validity and reliability for Chinese female college students, with Cronbach’s alpha levels of 0.92 for the Appearance subscale, and 0.94 for the Weight subscale [77]. In this study, the Cronbach’s alpha levels for the combined scale demonstrated strong reliability, with Cronbach’s alpha values of 0.92 at T1 and 0.91 at T2.

#### Eating behaviors

The Chinese version of the Three-Factor Eating Questionnaire-R21 were used [81]. Participants rated each item on a 4-point Likert scale, ranging from 1 (*definitely false*) to 4 (*definitely true*). Higher scores indicate greater problematic eating tendencies. An example item from the Uncontrolled Eating subscale is, “Sometimes when I start eating, I just can’t seem to stop.” An example item from the Emotional Eating subscale is, “I start to eat when I feel anxious.” An example item from the Cognitive Restraint subscale is, “I deliberately take small helpings as a means of controlling my weight.” This Chinese version has demonstrated good validity and reliability among Taiwanese students, with Cronbach’s alpha levels of 0.84 for Uncontrolled Eating, 0.91 for Emotional Eating, and 0.79 for Cognitive Restraint in the original study [81]. In the current sample, Cronbach’s alpha levels for Uncontrolled Eating were 0.81 at T1 and 0.86 at T2, Emotional Eating had alpha levels of 0.86 at T1 and 0.91 at T2, and Cognitive Restraint was 0.72 at T1 and 0.81 at T2.

#### Sociodemographic characteristics and BMI

Participants reported their age, weight (in kg), height (in meters), parental education level, annual family income (in RMB, the official Chinese currency), number of household members, Chinese ethnicity, hometown city, English language proficiency, study major, U.S. university, and prior travel to the U.S.

Socioeconomic status (SES) was calculated by averaging the standardized family income and the standardized parental education level (averaged between those of the two parents). BMI was calculated based on self-reported height and weight using the formula: body weight (in kg) divided by height (in meters) squared [82]. BMI was included as exploratory covariate given evidence that weight changes during the college transition are common [83] and may be related to body esteem and eating behaviors [84].

#### Free-response questions

Open-ended free-response items were included in the questionnaire at both T1 and T2 to capture participants’ perspectives beyond standardized measures. Participants provided written, text-based responses directly within the survey questionnaire. At T1, participants were asked: (1) “Regarding your body image, do you have any concerns or anything you would like to share?”; (2) “Regarding your eating behaviors, do you have any concerns or anything you would like to share?”; and (3) “Do you have anything else you would like to share?”

At T2, the same three open-ended questionnaire items were repeated with additional text prompts encouraging reflection on cultural transition following relocation to the United States. Specifically, participants were asked: (1) “Regarding your body image, do you have any concerns or anything you would like to share? (For example, after moving to the U.S., how does your perception of body image compare to when you were in China? )”; (2) “Regarding your eating behaviors, do you have any concerns or anything you would like to share? (For example, after moving to the U.S., how does your eating behavior compare to when you were in China? )”; and (3) “Do you have anything else you would like to share?”

### Analytical strategy

Quantitative analyses were conducted using SPSS (Version 24.0) on macOS. First, statistical assumptions were examined [85]. Missing data were minimal, with no missing values detected for variables collected at T1 and only two missing values for BMI at T2. Outliers were assessed based on three times the interquartile range (IQR = 2). One outlier was identified in the BMI variable at T2 (BMI = 33.66); however, this value fell within a reasonable range and was retained for analysis. All variables, except for BMI at T2, were normally distributed (|skewness| < 0.87, |kurtosis| < 0.89). The kurtosis level of the BMI variable at T2 was slightly high (kurtosis = 2.91) due to the identified outlier but was still within the normal range. Preliminary analyses included descriptive statistics and bivariate correlations to understand participant characteristics and relationships among study variables at both time points.

Group-level changes in the study variables from T1 to T2 (Study Aim 1) were examined using paired-sample *t*-tests. Cohen’s d was used to demonstrate effect size for the significant differences. Then, hierarchical linear regressions were conducted to examine whether acculturative stress explained variations in T2 variables beyond those measured at T1 (Study Aim 2). In Step 1 of the hierarchical regression models, the corresponding T1 variable (e.g., perceived pressures from family at T1) was entered, and in Step 2, acculturative stress (assessed at T2) was added to predict the T2 variable (e.g., perceived pressures from family at T2).

To examine participants’ perspectives on how cultural change influences body image and eating behaviors (Study Aim 3), participants’ free responses were analyzed using Codebook Thematic Analysis [86–88]. This method, positioned between codebook reliability and reflexive thematic analysis, is recommended for applied health research and was appropriate for the study because it accommodates both inductive identification of themes emerging from the data and deductive coding informed by the Tripartite Influence Model [20]. It further enabled analysis at both the manifest level (e.g., explicit references to body weight, food availability, peer comparisons) and the latent level (e.g., implicit tensions between Chinese and U.S. sociocultural norms). A preliminary codebook was developed collaboratively and refined iteratively during the coding process (e.g., initial codes such as “body ideal” and “food availability” were expanded into more nuanced categories such as “muscular and fit ideal” and “lack of vegetables” as analysis progressed). Two coders (P.W. and W.Z.) independently used the codebook, compared interpretations, and resolved discrepancies through discussion. Themes were then refined to ensure coherence, reduce conceptual overlap, and capture shared patterns of meaning both across participants and across time points, as well as within-participant changes over time.

### Positionality statement

In cross-cultural qualitative research, the inclusion of team members who can serve as cultural brokers is recommended to support culturally informed study design and interpretation [89]. The research team comprised investigators trained in psychology and related social sciences, with substantive expertise in body image, sociocultural processes, and cross-cultural research. Several authors are bilingual in Mandarin Chinese and English and have lived experience as international students or scholars, representing diverse cultural backgrounds beyond a single national context.

The primary author holds a PhD in psychology and brings lived experience as an international student and first-generation scholar, which informed the development of culturally relevant study materials and supported culturally grounded interpretation throughout data collection and analysis. The second coder, also bilingual in Mandarin Chinese and English, has formal training in qualitative data analysis and contributed to coding and analytic interpretation. Additional authors bring bicultural perspectives and decades of research experience, further strengthening analytic rigor. Three research assistants were international undergraduate students or individuals who had lived in both China and the United States for substantial periods and contributed linguistically and culturally informed perspectives.

Reflexive discussions were integrated throughout the analytic process to examine how researchers’ positionalities and assumptions might influence coding and interpretation. Coding decisions were reviewed collaboratively, and all authors contributed to interpretation of the findings and development of the implications.

## Results

### Participants’ characteristics

Among all freshman female international college students in the analysis, the average age at T1 was 18.29 years (*SD* = 0.51; range: 18–20 years old, Table 1). At T1, participants’ average BMI was 19.97 (*SD*_t1_ = 3.12) and at T2, participants’ average BMI was 20.60 (*SD*_t1_ = 3.01). The majority (*n* = 107, 96%) were from the Han ethnic background, and 57% had previously visited the U.S. On average, participants had visited four countries (*SD* = 3). Most enrolled at a university on the West Coast (*n* = 92), followed by the East Coast (*n* = 18) and the Midwest (*n* = 2). At T1, most participants rated their English proficiency as basic (*n* = 41), followed by limited working proficiency (*n* = 37), professional working proficiency (*n* = 26), full professional proficiency (*n* = 4), native/bilingual proficiency (*n* = 3), and not proficient (*n* = 1). By T2, most participants rated their English proficiency as limited working proficiency (*n* = 48), with others reporting basic proficiency (*n* = 26), professional working proficiency (*n* = 25), full professional proficiency (*n* = 6), native/bilingual proficiency (*n* = 6), and not proficient (*n* = 1). On average, their English proficiency ratings increased significantly (*p* = .01).

Regarding their field of study, most participants indicated Arts, Humanities, and Media (*n* = 19) or Undecided/Undeclared (*n* = 19), followed by Psychology and Cognitive Sciences (*n* = 17), Economics and Business (*n* = 15), Mathematics and Statistics (*n* = 14), Social Sciences (*n* = 10), Biological Sciences (*n* = 9), Environmental Sciences (*n* = 4), Computer Science and Engineering (*n* = 3), and Physical Sciences (*n* = 2). Geographically, most participants were from East China (*n* = 43), followed by North China (*n* = 38), South China (*n* = 14), Central China (*n* = 10), and West China (*n* = 6).

### Preliminary analysis

At both time points, higher body esteem was correlated with lower pressures from all four sources (family, peers, and significant others, as well as media), lower internalization of thinness and general attractiveness ideals, fewer problematic eating behaviors (uncontrolled eating, emotional eating, and cognitive restraint), lower BMI, and lower acculturative stress. At Time 1, body esteem was also correlated with lower internalization of muscular ideals (Table 1).

Across both time points, cognitive restraint was associated with higher pressures from all four sources and greater internalization of thinness and general attractiveness ideals. Uncontrolled eating was consistently related to higher acculturative stress. At Time 2, uncontrolled eating and emotional eating were additionally associated with higher pressures from all sources. Uncontrolled eating at Time 2 was also related to greater internalization of thinness and general attractiveness ideals. Finally, uncontrolled eating and emotional eating at Time 2 were related to higher BMI.

Because more than half of the participants had previously traveled to the U.S., all variables were examined by U.S. travel history to explore potential group differences. No significant differences emerged in most variables as a function of prior U.S. travel experience (*p*s > 0.06), except for the internalization of muscular ideal. At both time points, those who had been to the U.S. scored higher in the internalization of muscular ideal than those who had never been to the U.S. (T1: *M*_never been_ = 2.78, *SD* = 0.80; *M*_had been_ = 3.34, *SD* = 0.89, *t* [110] = -3.45, *p* = .001, d = − 0.66; T2: *M*_never been_ = 2.93, *SD* = 0.88; *M*_had been_ = 3.33, *SD* = 0.94, *t* [110] = -2.31, *p* = .02, d = − 0.44). Thus, U.S. travel history was included as a covariate in exploratory analysis.

### Main analysis

#### Paired sample T-test

At the group level, body esteem decreased (*t* = 2.19, *d* = 0.41), whereas emotional eating (*t* = -3.30, d = − 0.62), and BMI increased (*t* = -4.92, *d* = − 0.94; Table 1). Other variables did not show significant changes across time (*p*s > 0.28).

Exploratory repeated-measures ANCOVA, with time as the within-subject factor and U.S. travel history as the between-subject factor, showed that travel history did not moderate the decrease in body esteem or the increases in emotional eating and BMI. However, U.S. travel history moderated the change of pressures from family, peers and significant others, pressures from media, and cognitive restraint eating. Specifically, perceived pressures from family, peers and significant others increased from T1 to T2 among participants without U.S. travel history (before: *M* = 2.41, *SE* = 0.13; after: *M* = 2.66, *SE* = 0.13) but decreased among those with such experience (before: *M* = 2.52, *SE* = 0.11; after: *M* = 2.43, *SE* = 0.12; interaction: time x U.S. travel history *F* = 8.22, *p* = .01). Similarly, perceived pressures from media increased from T1 to T2 among participants without U.S. travel history (before: *M* = 3.52, *SE* = 0.17; after: *M* = 3.74, *SE* = 0.16) but decreased among those with such experience (before: *M* = 3.73, *SE* = 0.15; after: *M* = 3.53, *SE* = 0.14; interaction: time x U.S. travel history *F* = 6.19, *p* = .01). Finally, cognitive restraint eating increased from T1 to T2 among participants without U.S. travel history (before: *M* = 2.13, *SE* = 0.10; after: *M* = 2.26, *SE* = 0.11) but decreased among those with such experience (before: *M* = 2.27, *SE* = 0.08; after: *M* = 2.07, *SE* = 0.09; interaction: time x U.S. travel history *F* = 9.00, *p* = .003). Other changes in the variables were not moderated by U.S. travel history.

**Table 1:** Descriptives, bivariate correlations among study variables at each time point, and paired sample T-test results

	Descriptives statistics				Paired-sample t-test	Bivariate correlations											
	Time 1		Time 2														
Variable	*M*	*SD*	*M*	*SD*		1	2	3	4	5	6	7	8	9	10	11	12
1. Pressure from family, peers, and significant others	2.47	0.89	2.52	0.93	-0.90	–	**0.38** ^***^	**0.08**	**0.22** ^*^	**− 0.60** ^***^	**0.30** ^**^	**0.23** ^*^	**0.40** ^***^	**0.48** ^***^	**0.15**	**− 0.08**	**0.23** ^*^
2. Pressure from media	3.64	1.18	3.62	1.10	0.21	0.52^***^	–	**− 0.07**	**0.54** ^***^	**− 0.41** ^***^	**0.26** ^**^	**0.19** ^*^	**0.35** ^***^	**0.14**	**0.08**	**− 0.05**	**0.21** ^*^
3. Internalization of muscular ideal	3.10	0.89	3.16	0.89	-0.80	0.13	0.04	–	**− 0.16**	**− 0.25** ^**^	**0.05**	**− 0.01**	**− 0.05**	**0.09**	**− 0.16**	**0.01**	**0.15**
4. Internalization of thinness and general attractiveness ideal	3.86	0.62	3.87	0.61	-0.18	0.24^*^	0.47^***^	− 0.01	–	**− 0.37** ^***^	**0.20** ^*^	**0.05**	**0.30** ^**^	**− 0.12**	**0.04**	**− 0.01**	**0.09**
5. Body Esteem	2.35	0.70	2.26	0.68	2.19^*^	− 0.53^***^	− 0.49^**^	0.07	− 0.49^***^	–	**− 0.41** ^***^	**− 0.34** ^***^	**− 0.36** ^***^	**− 0.33** ^***^	**− 0.16**	**− 0.01**	**− 0.34** ^***^
6. Uncontrolled eating	2.23	0.52	2.24	0.60	-0.23	0.16	0.02	0.02	0.12	− 0.25^**^	–	**0.60** ^***^	**0.23** ^*^	**0.21** ^*^	**0.01**	**− 0.11**	**0.31** ^**^
7. Emotional eating	2.31	0.68	2.52	0.78	-3.30^**^	0.15	− 0.02	0.09	0.10	− 0.33^***^	0.49^***^	–	**0.34** ^***^	**0.23** ^*^	**0.03**	**0.02**	**0.02**
8. Cognitive restraint	2.21	0.67	2.15	0.75	1.08	0.35^***^	0.50^***^	0.08	0.46^***^	− 0.50^***^	0.05	0.15	–	**0.09**	**0.09**	**0.04**	**− 0.09**
9. BMI	19.95	3.14	20.60	3.01	-4.92^***^	0.47^***^	0.20^*^	− 0.04	− 0.14	− 0.34^***^	0.07	0.15	0.13	–	**0.00**	**− 0.12**	**− 0.01**
10. Age	18.29	0.51	18.76	0.70	-9.47^***^	− 0.04	− 0.01	− 0.04	0.08	− 0.09	− 0.09	− 0.02	0.16	–0.08	–	**0.01**	**0.15**
11. SES	0.01	0.57	–	–	–	0.06	0.16	0.18	0.06	− 0.03	0.04	0.03	0.09	− 0.10	0.01	–	**0.04**
12. Acculturative Stress	–	–	2.66	0.53	–	0.19^*^	0.20^*^	0.00	0.03	− 0.28^**^	0.24^*^	0.03	− 0.01	0.01	0.07	0.04	–

The bottom-left diagonal represents T1 correlations, while the top-right diagonal (bolded) represents T2 correlations. The diagonal entries indicate the correlations of the same variable between T1 and T2. A dash indicates that the test is not applicable

^*^p < .05, ^**^p < .01, ^***^p < .001

#### Hierarchical regression analysis

Acculturative stress at T2 was associated with greater internalization of the muscular ideal, lower body esteem, and higher uncontrolled eating (all at T2), after accounting for their T1 levels. For internalization of the muscular ideal, higher T1 scores were strongly related to higher T2 scores (*b* = 0.66, *p* < .001). Greater acculturative stress at T2 was associated with higher internalization of the muscular ideal at T2 (*b* = 2.56, *p* = .046, *β* = 0.15), while the association between T1 and T2 internalization remained significant (*b* = 0.66, *p* < .001). Acculturative stress accounted for an additional 1.7% of the variance (adjusted, *p* = .046).

For body esteem, higher T1 scores were associated with higher T2 scores (*b* = 0.75, *p* < .001). Greater acculturative stress at T2 was associated with lower body esteem at T2 (*b* = -0.17, *p* = .030, *β* = − 0.14), while the association between T1 and T2 body esteem remained significant (*b* = 0.71, *p* < .001). Acculturative stress explained an additional 1.4% of the variance (adjusted, *p* = .03).

For uncontrolled eating, higher T1 scores were related to higher T2 scores (*b* = 0.72, *p* < .001). Greater acculturative stress *at T2* was associated with higher uncontrolled eating at T2 (*b* = 0.20, *p* = .02, *β* = 0.17), while the association between T1 and T2 uncontrolled eating remained significant (*b* = 0.67, *p* < .001). Acculturative stress explained an additional 2.3% of the variance (adjusted, *p* = .020). Acculturative stress was not significantly associated with other variables at T2 after accounting for their T1 levels. These results remain when U.S. travel history was included as the covariate.

### Qualitative analysis

The qualitative data, at both time points, included written responses on body image (T1: *n* = 103; T2: *n* = 105) and eating behaviors (T1: *n* = 87; T2: *n* = 105) at T1Participants who did not contribute either skipped the open-ended items or indicated that they had “nothing to share.” Response length varied substantially, ranging from concise comments of four words to extended narratives of 304 words. Themes, selected examples, and their links to theory, quantitative findings or new insights are summarized in Table 2.

#### Change in body image

##### Theme 1: Adjustment to the U.S.: food, media, and internal conflicts

Exposure to calorie-dense foods alongside constant exposure to social media ideals intensified participants’ body awareness, leading some to experience conflicting emotions of dissatisfaction and pride toward their appearance. Several noted that the U.S. food environment made weight gain difficult to avoid. One remarked: “In China, I wasn’t too worried about my weight, but after coming to the U.S., the food made me gain weight.” Some described how stress and availability of food interacted to fuel body image concerns:

I gained a lot of weight after coming to the U.S., which made me anxious. Winter made it worse. Under exam pressure, I often binge eat and can’t control it, even though I know it’s unhealthy. In the dining hall, I always eat much more ice cream than others.

Social media remained an important influence. For some, platforms such as Xiaohongshu [RedNote, a social media platform popular in China] shifted their recommendations from thinness-oriented beauty standards toward muscular, fit oriented ideals:

I can clearly feel that even the content pushed to me on Xiaohongshu has changed, from the thin, delicate beauties I used to see to many videos of women building muscle and showing definition.

For others, the challenge was not simply external but lay in how social pressures were experienced and internalized across contexts. Even though people felt less openly judged in the U.S., their body image worries did not disappear. They now struggled to balance a more accepting culture with their own internal pressures. One woman reflected on the contradictions between what she saw around her and how she treated herself:

Although I believe we now advocate body freedom and oppose appearance anxiety, many times we can accept that others have body fat, but the one thing we can’t accept is that we ourselves have body fat. I still like the feeling of being slim, with a bit of muscular definition.

Another participant described a more positive trajectory, noting that greater diversity reduced her anxieties: “I felt there were many more people with a body type similar to mine, so I wasn’t as anxious anymore.” Still, pressures were not fully erased. Some participants highlighted that while the broader U.S. environment was more tolerant, social media, Chinese peers, and close friends continued to shape body image concerns:

After coming to the U.S., I encountered more diversity and inclusivity, but at the same time I still faced pressure from social media, Chinese classmates, and friends. They all influenced how I viewed my body image. But overall, I think my body image now is quite good, healthy, proportionate, and active, which is more important than chasing the so-called ‘pale, youthful, and thin look.’

##### Theme 2: Shift from thinness ideals to muscular/fit ideals

Another theme from participants’ narratives was a shift from thinness-centered ideals in China to fit- and body shape- oriented perspectives after exposure to U.S. cultural contexts. This transition again reflected both external sociocultural influences (e.g., media discourses, peer comparisons) and internal negotiations of self-concept. For instance, one participant explained that, although thinness continued to hold some value, her aesthetic preferences had broadened toward strength and muscularity: “Although I still retain some of the Chinese standards of beauty (not the extreme ‘pale, youthful, and thin’ look, just not being too fat), after coming here I lean more toward wanting to gain muscle. I think muscle definition is beautiful.” Another participant at T2 reported: “A fit body after working out really looks good. In China, it is rare to see such attractive bodies.”

At the same time, not all participants experienced this shift positively. One described the tension between aspiring toward a muscular, fit-oriented ideal and managing her own bodily changes:

When I first arrived, I thought I was too thin and assumed everyone liked people who were more muscular. Because I thought I couldn’t gain weight, combined with stress, I started eating without control, gained 10 kg, and now I have started dieting again.

##### Theme 3: Reduced anxiety and greater acceptance in the U.S

Many participants reported experiencing less body image anxiety and greater tolerance for diverse appearances after moving to the U.S. Shifts in cultural environment were often described as central to these changes, with participants linking greater inclusivity in U.S. settings to a sense of freedom in appearance management. For example, one participant described feeling self-conscious about her appearance prior to studying abroad: “Sometimes I feel anxious about my appearance” (T1). At the follow-up, the same participant emphasized that a more accepting cultural climate in the U.S. alleviated these concerns: “People are more inclusive about body appearance and thus my appearance anxiety has decreased” (T2). This pattern was echoed across participants. For instance, another woman explained: “Body anxiety is less in the U.S. I can wear whatever clothes I want. There hasn’t been any major change, but I feel much more freedom in how I dress.”

Cultural differences were frequently cited as an explanation for these shifts. One participant reflected: “I think due to the cultural difference, the U.S. is more accepting regarding weight. I definitely accept myself more because of that.” Another emphasized how diversity itself helped reduce social comparison pressures: “Because in the U.S., people come from different cultural and racial backgrounds, I experience much less anxiety about my body and appearance.” Similarly, a student contrasted her experiences directly: “When I was in China, people often thought I should lose weight. But in the U.S., no one has ever told me to diet.”

Importantly, participants also noted that this reduction in anxiety was context dependent. Anticipation of returning to China often reignited appearance concerns. As one participant explained: “When I plan to return to China in a month, I start to feel anxious about my body again. For example, I gained some weight in the U.S., and I worry whether my friends back home will think I look heavier.”

Some participants emphasized that studying abroad reduced the direct family pressures that had previously shaped their body image. For example, one explained while in China:

In the eyes of most Chinese parents, not wearing a bra is considered shameless, at least my mom said that. I was forced to start wearing one after some development, and I felt that if I didn’t wear it when going out with her, it would embarrass her, not me.

After moving to the U.S., she indicated “Now I am more certain that health matters more than body shape.” Another participant similarly described how maternal criticism once shaped her appearance concerns but gradually lost its influence:

A few years ago, my mother would PUA me [psychologically manipulate me], very directly pointing out what parts of my body didn’t look good and advising me to eat less. By the time I reached senior year of high school, I gradually realized the impact of this PUA and my own distorted thinking. Now, I increasingly like my body, regardless of whether it is fat or thin.

##### Theme 4: Cultural and practical differences in clothing norms

Clothing emerged as a salient domain in which participants recognized cultural differences in body image expectations. Size availability, labeling practices, and norms of dress freedom were frequently described as shaping perceptions of one’s body and self-confidence. Participants noted that clothing sizes in China were generally smaller, often leading to frustration or heightened body consciousness. One participant explained:

In China, sizes are generally small. For a woman taller than 170 cm, many clothes sold on Taobao have shoulders that are too narrow or bust sizes only in the seventies. A girl who is of normal weight and slightly curvy, but not overweight, often needs to buy XL in China. In the U.S., I can wear M instead, so it feels less restrictive, and I have more choices.

Another participant emphasized how the same body could be framed differently by clothing sizes across contexts: “In China, I felt my body was in the normal range, even considered desirable by some, and I usually bought M. But in the U.S., I buy S.” The symbolic impact of sizing differences was especially clear when participants contrasted how others treated them. One reflected: “…The tolerance for different body sizes is much higher. For example, in China I had to wear XXL, but in the U.S., L is enough.”

For some participants, these shifts in clothing fit were linked to increased self-acceptance over time. One participant described her own trajectory: T1: “I wasn’t very anxious, whether about body hair or body shape, and I was strongly against the cosmetic surgery trend.” Even though this woman felt relatively positive about their body image, at T2, she reflected “I feel more confident now. In China, I had to wear 4XL, but here XL is the largest I need. My aesthetic values have also become more diverse.” Another participant described a similar transition: T1: “I wanted to lose weight; my stomach was always big and my thighs thick, though other parts were fine. I didn’t dare to wear crop tops.” T2: “I don’t think I’m fat. In China, I was considered a little overweight and my mother often told me to lose weight. In China I wore L, but in the U.S., I can wear S or XS.”

##### Themes 5: Continuity of positive body image and dissatisfaction

Participants’ responses highlighted an ambivalent continuity in body image experiences across cultural contexts. While many women described growing confidence and increased self-acceptance over time, dissatisfaction and body-related concerns often persisted alongside these positive shifts. Rather than a linear progression toward positive body image, participants’ narratives reflected a nuanced balance between acceptance and continued vigilance.

For some participants, confidence and acceptance were continuous across time, reflecting resilience in navigating sociocultural pressures. One participant explained that she rarely experienced body image anxiety and opposed trends such as cosmetic surgery at T1, later noting at T2 that she felt “more confident.” Another reflected that, compared with earlier stages of life when she had endorsed items reflecting body-related anxiety, she now felt “mentally stronger and more satisfied,” and that her appearance concerns had gradually eased. Other participants articulated explicitly body-positive perspectives. One emphasized at T1 that women should “be confident regardless of being ‘fat, thin, tall, or short,’ refuse labels, and love themselves,” later adding at T2 that individuals must “learn to accept themselves despite the distorted aesthetics shaped by the internet.” Another commented at T1: “I really like my body!” The same participant later at T2 indicated: “I have become more tolerant of body image. Everyone has their own unique beauty, and there is no need to follow trends. Health itself is the most beautiful.”

In contrast, other participants reported that dissatisfaction persisted or intensified, despite greater exposure to inclusive cultural messages in the U.S. One participant described wanting a “perfect body to wear all the clothes I wanted” at T1 but noted at T2 that she still felt “very anxious about body shape when with other Chinese or Asian peers.” Another explained at T1: “I didn’t like my stretch marks or uneven skin.” Then at T2, she mentioned: “Even though I have kept exercising, I have developed more serious appearance and body anxiety. Maybe it’s because I have more friends around, and I can’t help but start scrutinizing myself.” Others described ongoing weight-focused concerns. One noted, “I was a little chubby and didn’t like my leg shape,” while aiming for a target weight of 50–55 kg at T1, then explained at T2 that she “gained 20 pounds in her first year of college.”

#### Change in eating behaviors

##### Theme 1: Binge eating and loss of control

Participants described experiences of binge eating, emotional eating, and a broader sense of losing control over food. These behaviors were often linked to stress, loneliness, and dissatisfaction with available food options in the U.S. For some, weekends became moments of overindulgence after a week of unsatisfying dining hall meals: “Because I don’t really like the dining hall food during the week, I’m more likely to overeat when I go out on weekends to make up for the lack of taste.” Others explicitly tied overeating to emotional coping. One participant explained:

After coming to the U.S., I ate a lot. At first it was stress and loneliness that made me eat this way. Now I have found more of a balance, and I haven’t kept gaining, but I did put on 40 pounds. In China, I had some depressive symptoms with loss of appetite, but in the U.S., it turned into binge eating.

Stressful circumstances often triggered episodes of overeating: “After coming to the U.S., I felt my diet became worse. Sometimes it was because the dining hall food wasn’t very good or healthy, other times it was because of stress-I would binge eat.” The structure of U.S. campus life and the abundance of food options also contributed to overeating. One participant noted: “I ate about 10% more at each meal because the dining hall food here was pretty good. At home, the food wasn’t very good, so in China I ate very little each day.”

Loss of control was a recurring theme, with participants describing frequent snacking, overeating between meals, and even engaging in compensatory behaviors: “I ate more, but White people’s food didn’t fill me up. After coming to the U.S., because the main meals weren’t satisfying, I often snacked a lot in between, and I couldn’t really control myself.” “It was harder to control myself here than in China. I often binged and even vomited.”

##### Theme 2: Adaptation, peer influence, and independence

Participants further described difficulty adjusting to new eating habits after moving abroad, highlighting how cultural adaptation, peer influence, and newfound independence disrupted previous routines. Many described the process of adjusting to U.S. food culture as frustrating and limiting. Common complaints included a lack of variety, smaller food ranges compared to China, and meals that felt less flavorful or satisfying. One explained:

Takeout food here is bad… eating has become more casual, not as rich and diverse as back home. The choices are fewer, and I end up eating more protein, cheese, and meat. Under stress, I order takeout. Overall, I eat more calorie-dense but less nutritious foods, with too much fat and carbs.

Another reflected on how their attitude toward food shifted over time:

After coming to the U.S., I ate less overall and lost interest in food, mainly because of the food itself. I also became less picky; before, I tried to eat well within what I could, now as long as food is edible, that’s enough.

For some, adaptation involved using food as a coping strategy. As one described: “Living abroad, with the time difference from my parents and the challenges of independence, I sometimes relied on sweets to relieve stress and worries.”

Peers played a powerful role in shaping eating patterns, often in ways that disrupted prior habits. The all-you-can-eat setting of dining halls encouraged more indulgent eating: “I ate more because eating with different peers made me gradually let go of dietary restrictions, especially with the all-you-can-eat dining hall.” Another participant reflected on how her roommate’s snacking habits altered her own behavior: “After coming to the U.S., my roommate’s eating habits were completely different. I never ate snacks in China, but my roommate only ate snacks. I developed the same habit, and it was very hard for me.”

Newly found independence was both liberating and destabilizing for many. Without parental oversight, participants gained greater autonomy over food choices. One explained: “In the U.S., my eating became more irregular. I eat whenever I want, snacks are easy to buy, and without parents controlling me, it feels free. If I want snacks, I just eat them.” Others described indulging in foods that were restricted at home. One participant explained how ice cream became part of her routine:

In China, my mother never let me eat ice cream because she thought it was too ‘cold’ for the body and I had a weak stomach, so I rarely ate it. But after coming to the U.S., I even eat it during my period.

Autonomy sometimes encouraged overeating and subsequent body concerns. As one participant noted:

I definitely ate more, maybe because the portions in the dining hall are small, so I often had many bowls. Sometimes I overate or even binged. Because my daily intake increased, I gained a lot of weight in the first semester, which made me anxious, so I started consciously reducing food intake and calories, something I rarely did in China.

##### Theme 3: Exposure to high-calorie, less nutritious food environments

Many participants also emphasized nutritional contrasts between the U.S. and China, describing campus food as calorie-dense and less nutritionally balanced. These perceptions reflected broader concerns about dietary acculturation and perceived declines in diet quality abroad. Students frequently noted that U.S. foods were overly salty, high in sugar, and lacking in vegetables, leading to both physical and psychological dissatisfaction.

Several participants contrasted the relative healthiness of their diets in China with the changes they experienced abroad: “It is not as healthy as in China. I like to eat vegetables, but in the U.S. diet the proportion of vegetables feels much lower.”

Living arrangements and meal plan requirements further constrained food choices. One participant explained:

I live in a school dormitory with a 7-day unlimited meal plan, so my options are limited to the dining hall. But many of the meals don’t suit Asian tastes. Desserts are too sweet, vegetables are too few, dishes are too salty, and this food structure has changed the diet I was used to. It led me to consume more sugar, salt, and carbs.

Others described how the lack of satisfying or familiar foods in the dining hall prompted compensatory snacking: “Because the dining hall food is not very good, meals don’t feel satisfying, so I need to eat snacks or ice cream and desserts to make up for the lack of enjoyment.” Fast food, fried items, and processed snacks were often identified as difficult to avoid: “There are more fried foods here, and since I don’t have a kitchen to cook for myself, I end up consuming more oil from the dining hall.” “I ate more junk food, like instant noodles.” “There is a lack of vegetables.”

At the same time, some participants noted a paradox: although dining hall food was often described as unbalanced or unappealing, it occasionally offered healthier options compared to what they ate in China:

Because I mainly eat in the dining hall, and it directly provides healthy vegetables, I actually eat more healthily here than in China. But the food is not as tasty, so when I crave something specific and it’s available, I try to satisfy myself.

##### Theme 4: Irregular eating patterns

Another recurring change in eating behaviors was a shift toward irregular meals and frequent skipping of main meals. Participants commonly contrasted their more structured eating schedules in China with the less predictable routines they adopted in the U.S. Several noted that while they maintained three meals a day in China, moving to the U.S. disrupted this rhythm: “In China, I ate three regular meals a day. After coming to the U.S., because I couldn’t get used to the food here, I went from three meals a day to just one… but my weight didn’t decrease.”

Time constraints associated with university life often contributed to irregular eating. One explained:

After starting college, because of class schedules, I often eat very late, and lunch just gets skipped.” Others described more extreme patterns, including restrictive eating practices: “After coming to the U.S., my diet structure changed. I ate fewer carbs, much more vegetables and fiber, and sometimes I even dieted, just one meal a day, only black coffee to drink.

For some, irregular patterns involved alternating between restriction and overeating: “I felt my diet became unhealthier here. During the day I eat very little, maybe because of my class schedule, but then at night I eat a lot. I know this pattern is unhealthy.” Also, late-night eating and reliance on processed foods were also frequently mentioned: “I ate more high-calorie food here, but I still tried to include vegetables. Still, my meals became unhealthy, sometimes only one meal a day, often instant noodles at night because the dining hall food was so bad.”

##### Theme 5: Mixed adaptations: health-oriented adjustments and challenges with U.S. food

Participants described a complex mix of adaptation strategies in response to the U.S. food environment. On one hand, many made deliberate health-oriented adjustments to counteract the prevalence of high-calorie and processed foods. On the other, they expressed ambivalence, simultaneously enjoying certain American staples while rejecting them due to taste preferences or health concerns.

Several participants emphasized conscious efforts to eat more nutritiously. They described choosing whole grains, vegetables, and fruits to balance what they perceived as a more calorie-dense food landscape: “The available food here is higher in energy, and there are more unhealthy options, so I purposely choose more whole grains, vegetables, and fruits to improve my diet.” Awareness of nutrition labels and ingredient lists was also heightened: “I eat too much sugar now, but on the other hand I focus more on reading ingredient lists. I also try to eat more fruits and vegetables.” For some, the dining hall provided opportunities to eat “cleaner” than they had at home in China, where meals often emphasized heavy meat dishes: “The food here is cleaner. Eating in the dining hall makes me feel healthier, and I even got used to eating salads. At home in China, meals were heavy with meat, which felt burdensome for my stomach.”

At the same time, participants acknowledged moments of indulgence and ambivalence. American foods such as pizza and hamburgers were often consumed with enjoyment but followed by concern about their nutritional value:

After coming to the U.S., I ate quite a lot of high-calorie, high-fat foods like hamburgers and pizza. Because I worried about the impact on my health, I forced myself to eat more fruits, vegetables, and salads, even though I don’t think they taste very good. I also take vitamin supplements.

**Table 2:** Themes from qualitative analyses with quotes and theoretical/quantitative connections

Change in body image	Example	Link to theory/quantitative findings/ new insights
1. Adjustment in the U.S.: Food, Media, and Internal Conflicts.	“In China, I wasn’t too worried about my weight, but after coming to the U.S., the food made me gain weight.”	Supports quantitative findings of decreased body esteem. Consistent with the Tripartite Influence Model, highlighting the role of sociocultural pressures, while also pointing to the U.S. food environment as an additional, culturally specific factor influencing Chinese international students’ body image.
2. Shift from Thinness Ideals to Muscular/Fit Ideals	“When I first arrived, I thought I was too thin and assumed everyone liked people who were more muscular. Because I thought I couldn’t gain weight, combined with stress, I started eating without control, gained 10 kg, and now I have started dieting again.”	Aligns with quantitative findings linking acculturative stress to stronger internalization of the muscular ideal, highlighting how stress can trigger both shifts in body ideals and maladaptive eating patterns.
3. Reduced Anxiety and Greater Acceptance in the U.S.	“Body anxiety is less in the U.S. I can wear whatever clothes I want. There hasn’t been any major change, but I feel much more freedom in how I dress.”	Although quantitative results showed decreased body esteem at the group level, this theme reflects greater personal freedom and reduced anxiety, underscoring individual variability in adjustment processes.
4. Cultural and Practical Differences in Clothing Norms	“…The tolerance for different body sizes is much higher. For example, in China I had to wear XXL, but in the U.S., L is enough.”	While quantitative results indicated a decrease in body esteem at the group level, this theme illustrates improved self-perception tied to cultural sizing norms, highlighting individual variability in adjustment.
5. Continuity of Positive Body Image and Dissatisfaction	“I really like my body!” (T1) / “I have become more tolerant of body image. Everyone has their own unique beauty, and there is no need to follow trends. Health itself is the most beautiful.” (T2, same participant)	Illustrates the multidimensional nature of body image. Suggests underlying stability in body appreciation and dissatisfaction.

Change In eating behaviors	Example	Link to theory/quantitative findings/ new insights
1. Binge Eating and Loss of Control	“After coming to the U.S., I ate a lot. At first it was stress and loneliness that made me eat this way. Now I have found more of a balance, and I haven’t kept gaining, but I did put on 40 pounds. In China, I had some depressive symptoms with loss of appetite, but in the U.S., it turned into binge eating.”	Aligns with the Escape Theory and quantitative findings that acculturative stress is linked to greater uncontrolled eating.
2. Adaptation, Peer Influence, and Independence	“Living abroad, with the time difference from my parents and the challenges of independence, I sometimes relied on sweets to relieve stress and worries.”	Supports the Tripartite Influence Model by demonstrating how eating behaviors are shaped by sociocultural relationships and stress, illustrating the multidimensional nature of eating.
3. Exposure to High-Calorie, Less Nutritious Food Environments	“It is not as healthy as in China. I like to eat vegetables, but in the U.S. diet the proportion of vegetables feels much lower.”	Highlights the importance of diet acculturation in shaping eating behaviors among international students.
4. Irregular Eating Patterns	“In China, I ate three regular meals a day. After coming to the U.S., because I couldn’t get used to the food here, I went from three meals a day to just one… but my weight didn’t decrease.”	Underscores diet acculturation as a key factor influencing eating patterns, even when changes in intake do not translate directly to weight outcomes.
5. Mixed Adaptations: Health-Oriented Adjustments and Challenges with U.S. Food	“The available food here is higher in energy, and there are more unhealthy options, so I purposely choose more whole grains, vegetables, and fruits to improve my diet.”	Reinforces that eating behavior is multidimensional, with evidence of individual differences in adaptation strategies.

## Discussion

This study is among the first to investigate body image and eating behaviors among an international migrant population using both quantitative and qualitative approaches. At the group level, female Chinese international students reported lower body esteem and higher emotional eating six months post-settlement compared to before migrating to the U.S. for college. Acculturative stress experienced six months after the move was associated with higher internalization of the muscular ideal, lower body esteem, and higher uncontrolled eating at follow-up, even after accounting for baseline levels. Complementing these findings, qualitative analyses provided a nuanced account of students’ lived experiences. For body image, themes reflected reduced anxiety and greater body acceptance in the U.S., a shift from thinness ideals to muscular/fit ideals, and more lenient expectations regarding clothing size. At the same time, new challenges emerged, including navigating the U.S. food environment, influences of media exposure, and conflicts in how social pressures were experienced and internalized across cultural contexts. Some patterns remained unchanged, with many students demonstrating a persistence of body dissatisfaction across contexts, while a smaller number showed a persistence of positive body image. For eating behaviors, salient themes included exposure to high-calorie and less nutritious food environments, irregular eating patterns, adjustments to food and culture, peer influence, and greater independence in eating choices. Students also described episodes of binge eating and loss of control, alongside health-oriented adaptations and mixed experiences with U.S. food environments.

As expected, quantitative data showed that body esteem was lower six months after arrival in the U.S. compared to before migration to the U.S. for college, and acculturative stress experienced after the move was associated with lower body esteem. Acculturative stress was also related to higher internalization of the muscular ideal six months after arrival in the U.S. Qualitative findings (Theme 2) further illustrated how participants recognized the muscular/fit ideal promoted within the U.S. cultural environment, even though group-level quantitative data did not show an overall increase in muscular-ideal internalization. Extending the Tripartite Influence Model [20], these results suggest that acculturative stress may contribute to body dissatisfaction through greater internalization of muscular ideals. This interpretation is supported by participants’ narratives. For example, some described initially perceiving themselves as too thin and assumed that U.S. peers preferred more muscular bodies. Unable to gain weight and experiencing stress, they reported engaging in uncontrolled eating, which led to significant weight gain and subsequent attempts at dieting. Although this participant also noted that women in the U.S. tend to exercise more, which made her feel healthier, her reflection illustrates how the U.S. emphasis on a lean and fit body ideal may simultaneously contribute to lower body esteem, particularly among those who are naturally thin. Additionally, the decrease in body esteem may also be linked to weight gain, consistent with the group-level increase in BMI and past research [90]. One participant reported gaining considerable weight accompanied by heightened anxiety. They described turning to binge eating and overeating during periods of exam stress and noted that, compared to the attention they received in China, the lower level of attention in the U.S. left them feeling disappointed and concerned about their body shape.

Notably, although a group-level decrease in body esteem was observed in quantitative data, individual trajectories varied. Body esteem was shaped not only by cultural transitions and acculturative stress but perhaps also by contextual factors such as clothing norms and personal circumstances. Some participants, for instance, highlighted the greater availability and inclusivity of clothing sizes in the U.S. compared to China (Theme 4), noting that this inclusivity improved their body image. Interestingly, such reductions in appearance-related anxiety were often context dependent, as anticipation of returning to China frequently reignited body concerns. Similar experiences of realization have been documented among Canadian cisgender women and women in Europe, highlighting empowering processes of developing adaptive cognitive strategies that support self-agency and positive body image [91, 92]. Future work should examine how cultural and contextual factors interact to shape heterogeneous body image trajectories, with particular attention to protective as well as risk processes.

Problematic eating, particularly emotional eating, was higher six months after settlement in the U.S. compared to before migration, based on the quantitative analysis. While the rise in emotional eating may reflect the immediate impact of migration, uncontrolled eating appeared more directly linked to acculturative stress at follow-up. This pattern is consistent with prior research on acculturation and problematic eating behaviors but provides a more nuanced understanding of specific processes. Emotional eating and uncontrolled eating reflect distinct mechanisms: emotional eating refers to eating in response to strong emotions such as stress or anxiety rather than hunger cues and does not necessarily imply overeating, whereas uncontrolled eating involves a loss of hunger cues and a tendency to eat beyond satiety, often arising from prolonged or unmanageable stress [93]. Some research further suggests that emotional eating may act as a precursor to overeating behaviors, which can be rooted in food addiction [94]. Taken together, these findings align with theoretical perspectives suggesting that when stress becomes chronic and exceeds coping resources, it can evolve into more severe patterns of problematic eating such as uncontrolled eating.

Qualitative findings provided additional context, highlighting both challenges and variability in students’ adjustment to the U.S. food environment, consistent with prior research [95–97]. Many participants described dissatisfaction with the limited variety and flavor of dining hall meals, which they linked to uncontrolled snacking, reliance on unhealthy foods, or binge eating during periods of heightened stress. Yet perspectives were not uniform. Some students emphasized positive aspects of the dining hall, such as greater access to vegetables and healthier options compared to meals at home. At the same time, even those who valued healthier choices acknowledged that the food was less appealing in taste, which sometimes prompted cravings and compensatory eating when preferred foods became available. Additionally, because many students were required to purchase meal plans and live in residence halls, the combination of constant food availability, newfound independence in food choices, and experiences of acculturative stress was described as contributing to the development of uncontrolled eating habits.

Also consistent with expectations, perceived media pressure did not change significantly across time in the quantitative data. Notably, the overall level of perceived media pressure was higher than that of family, friends, or significant others in this sample. This pattern is consistent with prior literature and underscores that media remains a striking and persistent force shaping young women’s body image perceptions [98]. Qualitative analyses provided additional nuance. Participants noted that social media platforms tailor content both to geographic location, reflecting different sociocultural preferences regarding ideal body image, and to individual interests, making it difficult to escape cycles of unrealistic ideals when exposed to glamorous and unattainable images of models or influencers. This sentiment aligns with prior evidence that social media algorithms track user behavior and selectively promote content based on engagement patterns [99, 100]. Importantly, the observation that content tailoring also reflects changes in geographic location extends prior research and highlights a novel mechanism through which media pressures may vary across cultural contexts. Together, these findings suggest that even when overall levels of perceived media pressure remain stable, the nature of content, and therefore the specific forms of body image pressure, may differ across sociocultural environments.

In contrast, other variables within the Tripartite Influence Model, such as internalization of thinness and general attractiveness ideals, as well as perceived pressures from family, peers, and significant others, did not change significantly over the six-month period. This stability is likely because these constructs are less sensitive to short-term shifts over such a time frame. Both quantitative and qualitative data indicated that thinness and general attractiveness ideals remained relatively stable, suggesting that the thinness ideal continues to exert influence, whether in the form of thinness alone or the combined thin-and-lean preference [37]. However, participants noted that family pressures were more distal with greater independence, suggesting potential changes over a longer period (Theme 3, Change in Body Image). Peer influence appeared more complex, depending on whether peers were from the same ethnic background or from other racial and ethnic groups with differing cultural expectations (Theme 5, Change in Body Image). Pressures from significant others may also shift as students navigate new relationships during cultural transition, although this source of pressure was less frequently mentioned. Preliminary analyses further suggested that U.S. travel history may serve as a protective factor. Students with prior U.S. experience reported lower perceived pressures from family, peers, significant others, and media compared to those visiting the U.S. for the first time. This finding is consistent with prior research showing that independent self-construal can buffer against sociocultural stressors related to eating outcomes [101]. U.S. travel history may also index greater socioeconomic resources or prior exposure to Western cultural and media environments, which could facilitate critical engagement with appearance-related messages and protect against increases in appearance pressures and cognitive restraint eating over time. Nonetheless, the protective role of U.S. travel history did not extend to body esteem, reinforcing the idea that additional and more complex factors [e.g., internalization of different ideals, upword and downword appearance comparison; 20] may shape body esteem outcomes.

### Future direction

This mixed-methods study offers novel insights into group-level changes in body image and eating-related constructs among female Chinese international students, while providing rich qualitative context within the Tripartite Influence Model. Future research can extend these findings in several ways. First, because migration and college transition in this population co-occur and both entail significant social and environmental changes, future studies could incorporate domestic comparison groups to disentangle migration-specific from more general developmental influences on body image and eating behaviors. Second, findings indicate that geographic location may influence media-related pressures through algorithm-driven content personalization. Future studies should examine how regional digital environments interact with personalized media exposure to shape body image concerns, an area that remains largely unexplored. Finally, declines in body esteem may be partially linked to weight changes and stress-related eating patterns. Multi-wave longitudinal designs with adequate statistical power are needed to test this potential mechanism and explore the moderating role of body weight.

### Limitations

Several limitations should be considered. First, while capturing experiences both before migration and after arrival is an important strength, two waves of data collection limit the ability to model more complex longitudinal processes. Future research with at least three waves of data could apply approaches such as the random-intercept cross-lagged panel model [102] to better distinguish within-person effects from between-person associations. Second, the absence of a control group of female Chinese students who remained in China means that the extent to which changes in the variables reflect cultural transition versus general time-related effects cannot be fully disentangled. Including such a control group in future studies would help clarify the unique role of cultural transition in shaping these outcomes. Future studies should also explore a broader range of eating behaviors, such as thinness-oriented or muscularity-oriented disordered eating or healthy aspects of eating such as mindful eating or intuitive eating.

Finally, participants were self-selected international first-year college students from mainland China, and recruitment relied on online outreach, which may have introduced selection bias. Consistent with the study’s inclusion criteria, individuals with a current or prior clinical diagnosis of an eating disorder were excluded. However, the absence of a clinical diagnosis does not preclude the presence of subclinical eating-related symptoms that may not have been formally assessed in clinical settings, particularly given that mental health stigma remains prevalent in many Chinese cultural contexts and may discourage help-seeking [103]. Accordingly, the findings should be interpreted as reflecting body image- and eating-related processes in a non-clinical, prevention-oriented context, rather than as characterizing clinically diagnosed eating disorder populations. Furthermore, acculturative stress may manifest differently across populations and stages of migration. For example, experiences of racial discrimination were not explicitly reported as a salient risk factor in the present qualitative findings but were endorsed at a moderate level in the quantitative assessment. Accordingly, the extent to which these findings generalize to other migrant populations and to individuals of diverse gender identities remains to be determined.

### Implications

Theoretically, extending the Tripartite Influence Model and broader sociocultural models of body image such as the Sociostructural-Intersectional Body Image framework [13], the results identify acculturative stress as a distinct sociocultural factor associated with body image and eating behaviors in international student populations. In addition, theories of acculturative stress may be strengthened by incorporating the concept of diet acculturation [104] as a central component, given that dissatisfaction with unfamiliar food contexts was linked to maladaptive eating patterns in both this study and prior research [64, 95].

Empirically, the observation that some participants demonstrated body positivity and resistance to prescriptive appearance ideals underscores the importance of identifying factors that predict greater body satisfaction or rejection of harmful media ideals. Also, while some aspects of cultural transition (e.g., acculturative stress) may exacerbate body image and eating concerns, other aspects such as increased autonomy, exposure to alternative value systems, and opportunities for critical reflection on appearance norms [91, 92], may function as protective or empowering influences that warrant future examination.

Practically, international students constitute a substantial proportion of university communities, and their body image and eating concerns warrant greater institutional attention. Although often perceived as relatively privileged, access to overseas education does not necessarily reflect broader economic security or reduced risk for eating disorders and body dysmorphic concerns. Rather, evidence from Chinese and Asian American populations suggests that higher socioeconomic status may be associated with elevated risk for body image concerns and disordered eating, potentially through heightened achievement pressures and sociocultural expectations [105, 106]. Accordingly, university dining services should offer more diverse and culturally sensitive meal options to reduce dissatisfaction with food environments and to support the academic success and retention of these students [107]. Incorporating evidence-based prevention and early intervention programs, such as self-compassion approaches delivered through guided meditations or mobile applications [108], may be particularly beneficial when tailored to international student populations. In contexts characterized by anti-immigrant and xenophobic rhetoric, interventions that both alleviate acculturative stress and address structural and interpersonal xenophobia may hold promise for international students. For example, cross-cultural centers [109] and culturally responsive pedagogy [110] that emphasize the visibility and celebration of diverse cultural values may be associated with greater campus-wide retention and intercultural competence, while potentially reducing international students’ exposure to xenophobic messaging and related distress. Universities and international student offices can play an important role in ensuring access to such resources and embedding them within broader wellness initiatives and orientations. Counselors should also remain attentive to the unique challenges these students face in balancing cultural expectations, stress, and eating habits. Training for counselors should include culturally responsive approaches that address acculturative stress (e.g., the recognition and assessment of race-based traumatic stress [111]), food environment adjustment, and body image concerns specific to international student populations.

At a broader level, these prevention-oriented findings, together with prior research documenting elevated eating disorder risk among migrant populations [112, 113] and international students from diverse national backgrounds studying in non-U.S. host contexts [114, 115], contribute to global discussions on migrant health and well-being. Policy efforts should prioritize cross-cultural health promotion, accessible counseling services, and food environment adaptation as part of comprehensive strategies to promote health and resilience among migrant populations in multicultural societies.

## Data Availability

The deidentified quantitative data supporting the findings are publicly available online: https://osf.io/gkhw3/overview? view_only=4ac9a6da4f944ccab2bfbf9f0e788ec3.
